# Once-daily fluticasone furoate/vilanterol 100/25 mcg versus twice daily combination therapies in COPD – mixed treatment comparisons of clinical efficacy

**DOI:** 10.1186/s12931-015-0184-8

**Published:** 2015-02-15

**Authors:** Gillian Stynes, Henrik Svedsater, Jaro Wex, Sally Lettis, David Leather, Emanuela Castelnuovo, Michelle Detry, Scott Berry

**Affiliations:** Value Evidence and Outcomes, GlaxoSmithKline, Stockley Park, London, UK; Clinical Statistics and Programming, GlaxoSmithKline, Stockley Park, London, UK; Respiratory Medicines Development Centre, GlaxoSmithKline, Stockley Park, London, UK; Health Investment Evidence, Global Health Outcomes, GlaxoSmithKline, Stockley Park, London, UK; Berry Consultants LLC, Austin, TX USA

**Keywords:** COPD, Fluticasone furoate, ICS/LABA, Mixed treatment comparison, Network meta-analysis, Vilanterol

## Abstract

**Background:**

Fluticasone furoate (FF)/vilanterol (VI) 100/25 mcg is a once-daily inhaled corticosteroid (ICS)/long-acting beta_2_ agonist (LABA) treatment approved in the United States, Canada and Europe for the long-term maintenance therapy of COPD. We report data from mixed treatment comparisons (MTC) of once-daily FF/VI against established twice-daily ICS/LABA combination therapies on clinical efficacy outcomes.

**Methods:**

Data from 33 parallel-group randomised controlled trials (RCTs) of ICS/LABAs, of ≥8 weeks’ duration in patients ≥12 years of age with COPD, identified by systematic review, were analysed using covariate-adjusted Bayesian hierarchical models for three efficacy outcomes. Lung function, assessed by change from baseline in forced expiratory volume in one second (FEV_1_), was the outcome of primary interest (n = 28 studies). Secondary objectives were assessment of annual rate of moderate/severe exacerbations (n = 15) and patient-reported health status, measured by change from baseline in St George’s Respiratory Questionnaire (SGRQ) Total score (n = 20). Overall, 25 different treatments were included in the MTC; we report findings, including probabilities of non-inferiority, for comparisons of once-daily FF/VI 100/25 mcg with twice-daily fluticasone propionate (FP)/salmeterol (SAL) 500/50 mcg and budesonide (BUD)/formoterol (FORM) 400/12 mcg.

**Results:**

For FEV_1_, FF/VI 100/25 mcg demonstrated >99% probability of non-inferiority to FP/SAL 500/50 mcg and BUD/FORM 400/12 mcg using a 50 mL margin. For annual rate of moderate/severe exacerbations, FF/VI 100/25 mcg demonstrated 73% and 77% probability of non-inferiority to FP/SAL 500/50 mcg and BUD/FORM 400/12 mcg, respectively, using a 10% rate ratio margin. For SGRQ Total score, the corresponding probabilities of non-inferiority were 99% and 98%, respectively, on a 2-unit margin. Significant covariate effects were identified: increased age was associated with deterioration in FEV_1_ and reduced exacerbation frequency; shorter study duration was associated with reduced exacerbation frequency.

**Conclusions:**

FF/VI 100/25 mcg was comparable with corresponding doses of FP/SAL and BUD/FORM on lung function and health status outcomes. Non-inferiority on moderate/severe exacerbation rate was not demonstrated to the same degree of confidence, though observed rates were similar. Model limitations include a weak treatment network for the exacerbation analysis and variability across the included studies. Our data support previous RCT findings suggesting that the efficacy of FF/VI 100/25 mcg on lung function and health status in COPD is comparable with twice-daily ICS/LABAs.

**Electronic supplementary material:**

The online version of this article (doi:10.1186/s12931-015-0184-8) contains supplementary material, which is available to authorized users.

## Introduction

Fluticasone furoate (FF)/vilanterol (VI) is an inhaled corticosteroid (ICS)/long-acting beta_2_ agonist (LABA) combination maintenance therapy, approved in 2013 in the United States, Canada and Europe at a strength of 100/25 mcg (equivalent to an emitted dose from the inhaler of 92/22 mcg) for chronic obstructive pulmonary disease (COPD). Unlike established ICS/LABA combination therapies, the 24-hour activity of both of its components means that FF/VI is suitable for once-daily dosing. Systematic reviews have supported the long-term use of LABAs and ICS/LABA combination therapies in COPD [1,2]. In randomised controlled trials (RCTs) in patients with COPD, FF/VI has consistently demonstrated an acceptable safety profile, similar to that of the comparable strength of the established twice-daily combination therapy fluticasone propionate (FP)/salmeterol (SAL), and been found to be well tolerated [3,4].

Several RCTs have examined the efficacy of FF/VI vs its components [5], placebo and components [6,7], placebo [8,9], tiotropium [10] or FP/SAL [3] in COPD. In 12-week double-blind, double-dummy head-to-head studies, the efficacy of once-daily FF/VI 100/25 mcg in improving lung function and health status in patients with COPD was shown to be similar to that of twice-daily FP/SAL 500/50 mcg [3,4]. The efficacy of once-daily FF/VI has not, at the time of writing, been directly compared in an RCT with that of ICS/LABA combination therapies other than FP/SAL, such as budesonide (BUD)/formoterol (FORM) 400/12 mcg (equivalent to an emitted dose of 320/9 mcg). However, the observed difference in efficacy between FF/VI and VI in patients with COPD is consistent with that reported between other ICS/LABA combinations and their LABA monocomponent [5,11,12], suggesting comparability of FF/VI with other ICS/LABA combination treatments in COPD.

We sought to investigate the relative treatment efficacy of FF/VI 100/25 mcg in COPD compared with alternative licensed ICS/LABA combination therapies, using a mixed treatment comparison (MTC) approach. This model-based methodology provides a means of estimating the relative efficacy of treatments that have not been directly compared in an RCT, and broadens the evidence base for those treatments which have already been compared in head-to-head studies. We conducted an MTC utilising a Bayesian, hierarchical model, combining data from separate RCTs identified through a systematic literature review to make inferences about the relative treatment efficacy of FF/VI 100/25 mcg compared with FP/SAL 250/50 mcg and BUD/FORM 400/12 mcg. RCTs included in the model involved a range of comparators; all included at least one ICS/LABA therapy. Three clinically relevant outcomes were examined: lung function as assessed by forced expiratory volume in one second (FEV_1_), exacerbations and health-related quality of life (Jansen, 2008). The primary focus of the analyses was on non-inferiority.

## Methods

### Systematic literature review

A systematic literature review was conducted to identify Phase III and Phase IV parallel-group RCTs of any ICS/LABA maintenance therapies vs any drug comparator(s). Studies of >8 weeks duration, in ≥10 patients aged ≥12 years, with an established diagnosis of COPD at any severity warranting treatment (defined as % predicted forced expiratory volume in one second [FEV_1_] ≤80%), who were receiving ICS or ICS/LABA maintenance therapy at randomisation, were included. Studies examining only short-acting beta agonists or short-acting muscarinic antagonists aimed at symptom control were excluded. RCTs of FF/VI were identified internally using the same criteria.

Studies were identified through the systematic searching of clinical publication databases and clinical trial registers (Additional file 1). Additionally, references in retrieved articles and relevant systematic reviews were checked for further studies that might fulfil the inclusion criteria. No date limits were applied to the searches.

### Outcome assessment

The outcomes assessed were: change from baseline in FEV_1_ (the outcome of primary interest); annual rate of moderate exacerbations (worsening symptoms of COPD that required treatment with oral corticosteroids and/or antibiotics) or severe exacerbations (worsening symptoms of COPD that required an emergency room visit and/or in-patient hospitalisation); and change from baseline in St George’s Respiratory Questionnaire (SGRQ or SGRQ-C) Total score.

The effect of treatment on lung function was assessed through reporting of change from baseline in FEV_1_. Because of the widely-accepted clinical importance of airflow limitation in COPD [13,14], FEV_1_ was considered the outcome of primary interest from the MTC. Exacerbation rates and SGRQ (a health status questionnaire with three components: symptoms, activity and impact on daily life) were the other outcomes of interest.

For each outcome, studies identified through the systematic literature review were included in the MTC if they reported the precise endpoint or sufficient calculable information in a suitable format. For exacerbations, the reporting of either the rate or number of moderate/severe exacerbation [15], in combination with number of patients, study duration and (for number of exacerbations only) number of withdrawals, was required for inclusion in the analysis. All treatment arms in each included study were included in the MTC, with one exception where data provided for the placebo arm were insufficient for the study’s inclusion in the exacerbations analysis [16].

A range of non-inferiority margins for each outcome were chosen on the basis of prior comparative studies in COPD. Margins for change from baseline in FEV_1_ and in SGRQ Total score represent approximately half of the accepted minimum clinically important difference (MCID) of approximately 100 mL and 4 units, respectively [17,18], and both the 2- and 3-unit thresholds were also examined for SGRQ Total score. For exacerbations, annual rate reduction margins of 10% and 20% were examined.

Exacerbation history is a very strong predictor of an individual’s risk of future exacerbations [19]. As such, to maximise comparability, the main analysis of exacerbation rate data was performed using data from only those studies that required an explicit history of exacerbation for study entry.

### Modelling strategy

The MTC modelling approach used for evidence synthesis accounts for variability across studies through parameterisation of the study effect to estimate relative treatment effects. The Bayesian approach [20-22] was decided upon prior to commencement of the systematic literature review. This methodology allows for inference from weak or disconnected treatment networks [23].

For each of the three outcomes, a hierarchical model was created whereby the effect of each included study *α* was modelled with a distribution *α*_*S*_ ~ *N*(*μ*, *τ*^2^). The two parameters *μ* and *τ* were then modelled with second-level hyperpriors and a posterior distribution created. Treatment effects were then modelled separately as single parameters with independent prior distributions, enabling the derivation of probabilistic comparisons between treatments together with credible intervals (CrI) for the differences in effect sizes.

For continuous outcomes (change from baseline in FEV_1_ and SGRQ Total score), the mean treatment effects were modelled with Normal distributions: *Y* ~ *N*(*α*_*s*_ + *θ*_*t*_ + *βZ*, *σ*^2^) with non-informative prior ${\sigma}_{\alpha}^2\sim Inverse- Gamma\left(0.001,0.001\right)$ and hyperpriors *μ*_*s*_ ~ *N*(0, 10^2^) and *τ*^2^ ~ *Inverse* − *Gamma*(0.001, 0.001). The parameters *α* and *θ* represent, respectively, the studies included in the analysis, and the treatment regimen effects. The *Z*’s represent the covariates and the *β*’s represent the coefficients (covariate effects). Each treatment effect was modelled independently with the flat prior distribution *N*(0, 100^2^).

Annual moderate/severe exacerbation rates were modelled using a Poisson distribution: Exac ~ Poisson(Rate*person-years), in which log(*rate*) = *α*_*s*_ + *θ*_*t*_ + *βZ*, requiring input of the number of moderate/severe exacerbation events and the number of person-years of follow-up. Priors and model parameters for study effects and treatment effects for this outcome were defined as for the other three outcomes, as was the distribution of study effects, with hyperpriors *μ*_*s*_ ~ *N*(0, 10^2^) and *τ*^2^ ~ *Inverse* − *Gamma*(0.001, 0.001).

Exacerbation rate was defined as the number of exacerbation events divided by person-years of follow-up. Person-years of follow-up were computed directly if both the rate and number of events were available or, if neither were available, the person-years were estimated. When estimated, patients not lost to follow-up were assumed to have had complete (100%) follow-up. Patients lost to follow-up were assumed to have 50% of the possible follow-up. When the number of events was not reported, the rate and estimated person-years of follow-up were used to estimate the number of moderate/severe exacerbation events. The priors and hyperpriors were similar to the other analyses.

Covariates were included in the models using a fixed-effects approach; a coefficient was created for each covariate. The coefficients were modelled independently with each having a flat prior distribution of N(0,10^2^). The following covariates were included in the ‘full-covariate’ models: study duration, age, gender, smoking status, percent predicted FEV_1_ and exacerbation history. The continuous covariates – age, gender and smoking history – were normalised. Specifically, age was normalised by subtracting 60 years of age; the resulting covariate is “Age – 60”. Gender is represented as the percentage of males in a treatment arm. Gender was normalised by subtracting 75% from the treatment arm population of males, thus the covariate is “proportion male – 0.75”. Smoking status is represented as the percentage of current smokers in a treatment arm. Smoking status was normalised by subtracting 50% from the treatment arm population of current smokers, thus the covariate is “proportion smokers – 0.5”. The categorical covariates – % predicted FEV_1_ at baseline, exacerbation history and study length – had designated reference groups of mean % predicted FEV_1_ 50– ≤ 70%; ≥1 exacerbation in previous year; and study duration 40–60 weeks, respectively. Findings from a reduced model including only one covariate (study duration) are provided in Additional file 1: e-Table S4.

For each outcome, model fit was evaluated by assessing the standardised residual, i.e. the difference between the model-estimated values and observed values divided by the estimated standard deviation.

All analyses were conducted using standard Markov Chain Monte Carlo methodology, utilising adaptive Metropolis-Hastings steps [24] where applicable, and were performed using custom software written in ANSI-standard Fortran (Berry Consultants LLC, Austin, TX). The software used was independently validated with duplicate code written in R (R Development Core Team, Vienna, Austria).

### Sensitivity analysis

A sensitivity analysis in which studies that were excluded from the primary analysis were added to the model was carried out for the annual moderate/severe exacerbation rate outcome. Six additional studies in which there were no explicit requirements for a history of patient exacerbations were included in the sensitivity analysis network. A second sensitivity analysis of the exacerbation rate data used a subset of the network including only those studies for which reported exacerbation rates were adjusted for follow-up. For the SGRQ outcome, one sensitivity analysis was performed where two studies were excluded from the network: one was removed because data values were markedly different from those in other studies in the model, and the other study was removed because baseline SGRQ values were much higher than for other studies in the model.

### Assessment of alternative modelling approaches

Two post-hoc analyses using alternative modelling approaches were conducted in order to evaluate the extent to which outcomes were susceptible to the primary model chosen. The same input data sets were used in all analyses. One set of analyses utilised the frequentist approach using a random effect model with fixed study and treatment effects and random study x treatment interaction. R software (*lme4* package) was used for effect estimation with *lmer* function used for FEV_1_ and SGRQ and *glmer* for exacerbations. The second set of analyses was based on pairwise contrasts, but with no covariate adjustment, and conducted using a Bayesian random effects model in geMTC software [25] running WinBUGS [26]. As geMTC did not enable automated analyses of rates with Poisson distributions, exacerbation rates were approximated as continuous variables, comparing rate differences rather than rate ratios. In the validation analyses, point estimates with confidence or credible intervals were calculated, with the frequentist analyses also reporting p-values.

## Results

### Study selection

**Figure 1:**
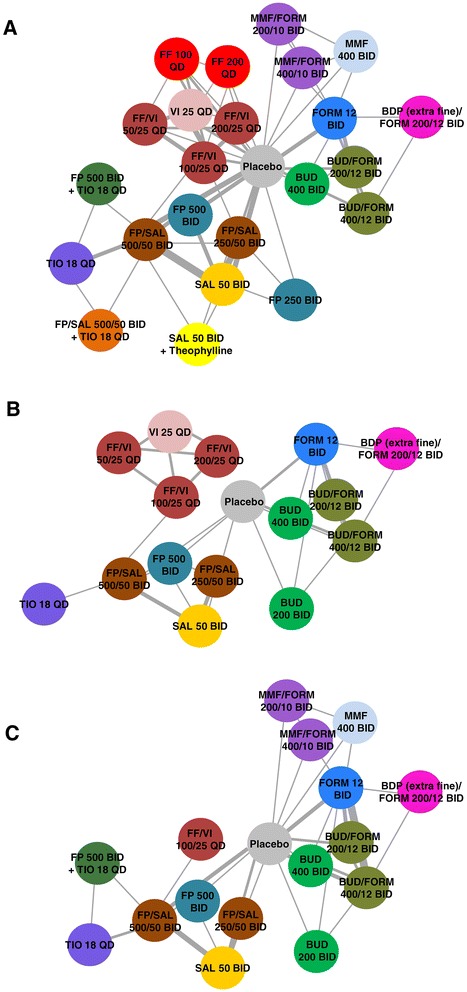
**Network of treatments connected by studies for each outcome of interest. A**: change from baseline FEV_1_, L; **B**: annual rate of exacerbations; **C**: change from baseline SGRQ Total score. *Note:* All stated nominal doses are mcg. Connecting lines represent studies included in the model that directly compare the two treatments. The thickness of the line is proportional to the number of studies comparing the two treatments. BDP = beclomethasone dipropionate, BID = twice daily, BUD = budesonide, FORM = formoterol, FF = fluticasone furoate, FP = fluticasone propionate, MMF = mometasone furoate, QD = once daily, SAL = salmeterol, THEO = theophylline, TIO = tiotropium, VI = vilanterol.

Fifty-nine unique studies were considered for inclusion in the MTC (Figure 1). A total of 33 trials were included in the primary analysis: of these, 28 were included in the analysis of the outcome of primary interest of change from baseline in FEV_1_; 20 were included in the SGRQ analysis; and 15 were included in the exacerbation rates analysis (Additional file 1: e-Table S1). The studies and treatment arms included in the MTC are summarised in Table 1. Reasons for exclusion of studies from each analysis are outlined in the Additional file 1: e-Appendix.

**Table 1:** **Summary of studies and treatment arms included in the mixed treatment comparison analysis** (**primary analysis**)

	**N (%)**		**N (%)**
**Total studies**	**33**	**Total treatment arms**	**104**
Endpoint reported		Treatments	
Change from baseline in FEV_1_	28 (85)	Placebo	14 (13)
Annual rate of moderate/severe exacerbations	15 (45)	FF/VI 50/25 QD	3 (3)
Change from baseline in total SGRQ	20 (61)	FF/VI 100/25 QD	7 (7)
Exacerbation history		FF/VI 200/25 QD	3 (3)
At least 1	14 (42)	FF 100 QD	2 (2)
At least 2	3 (9)	FF 200 QD	1 (1)
Not reported	16 (48)	VI 25 QD	4 (4)
% Predicted FEV_1_		FP/SAL 250/50 BID	8 (8)
<50%	13 (39)	FP/SAL 500/50 BID	14 (13)
50%–70%	16 (48)	FP/SAL 500/50 BID + TIO18 QD	1 (1)
>70%	4 (12)	SAL 50 BID	10 (10)
Mean age reported	63.79	SAL 50 BID + theophylline	1 (1)
Mean proportion male	0.73	FP 250 BID	1 (1)
Mean proportion current smokers	0.46	FP 500 BID	3 (3)
		FP 500 BID + TIO 18 QD	1 (1)
		BUD/FORM 160/9 BID	3 (3)
		BUD/FORM 400/12 BID	9 (9)
		BUD 160 BID	1 (1)
		BUD 320 BID	3 (3)
		FORM 9 BID	8 (8)
		MMF 400 BID	1 (1)
		MMF/FORM 200/10 BID	1 (1)
		MMF/FORM 400/10 BID	1 (1)
		TIO 18 QD	3 (3)
		BDP(extra fine)/FORM 200/12 BID	1 (1)

*Note:* All stated nominal doses are mcg.

*BDP* = beclomethasone dipropionate, *BID* = twice daily, *BUD* = budesonide, *FORM* = formoterol, *FEV*_1_ = forced expiratory volume in one second, *FF* = fluticasone furoate, *FP* = fluticasone propionate, *MMF* = mometasone furoate, *QD* = once daily, *SAL* = salmeterol, *SGRQ* = St George’s Respiratory Questionnaire, *TIO* = tiotropium, *VI* = vilanterol.

### Comparison of clinical efficacy of FF/VI with twice-daily ICS/LABA combinations

#### Change from baseline FEV_1_

**Figure 2:**
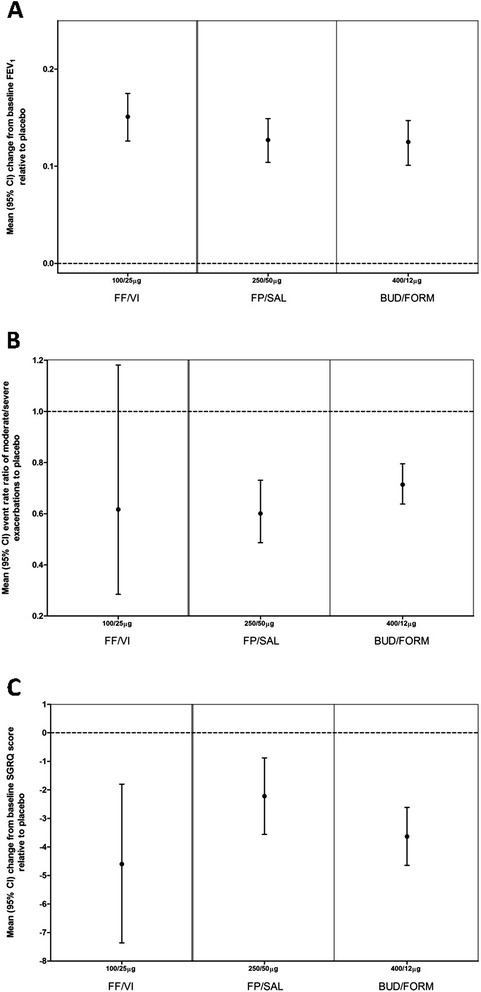
**Change from baseline versus placebo estimated using the full covariate model for selected treatments. A**: FEV_1_; **B**: rate ratio of moderate/severe exacerbations*; **C**: Total SGRQ. *Derived from studies in which patients were required to have an explicit exacerbation history at baseline. BUD = budesonide, CI = confidence interval, FEV_1_ = forced expiratory volume in one second, FF = fluticasone furoate, FORM = formoterol, FP = fluticasone propionate, SAL = salmeterol, SGRQ = St George’s Respiratory Questionnaire, VI = vilanterol.

FF/VI 100/25 mcg once daily was associated with an estimated mean (±SD) 28 ± 38 mL absolute improvement from baseline, higher than the estimated mean of 5 ± 40 mL for FP/SAL 500/50 mcg twice daily and the estimated mean of 1 ± 42 mL for BUD/FORM 400/12 mcg twice daily (Additional file 1: e-Table S2). On average, a decrease from baseline (−123 ± 39 mL) was seen with placebo. Hence, all three selected ICS/LABA combination therapies produced improvements in FEV_1_ vs placebo that exceeded the MCID of 100 mL [17] (Figure 2).

Based on a non-inferiority margin of approximately half the MCID (i.e. 50 mL), FF/VI 100/25 mcg demonstrated >99% probability of non-inferiority to both FP/SAL 500/50 mcg and BUD/FORM 400/12 mcg (Table 2). A significant covariate effect of age was seen; therefore a smaller change from baseline in FEV_1_ can be expected in studies enrolling older patients on average (Additional file 1: e-Table S3).

**Table 2:** **Posterior probability of non**-**inferiority for FF**/**VI 100**/**25 mcg versus other relevant ICS**/LABA* (**full covariate model**)

**A: change from baseline FEV**_**1**_				
**Treatment**	**Comparator**	**Mean difference, L (95% CrI)**	**Probability of non-inferiority margin** **(change from baseline)**	
			**50 mL**	
FF/VI 100/25	FP/SAL 500/50	0.023 (−0.002, 0.048)	>99%	
FF/VI 100/25	BUD/FORM 400/12	0.027 (−0.007, 0.061)	>99%	
**B: annual rate of moderate/severe exacerbations** ^**†**^				
**Treatment**	**Comparator**	**Rate ratio (95% CrI)**	**Probability of non-inferiority margin (event rate ratio)**	
			**0.10**	**0.20**
FF/VI 100/25	FP/SAL 500/50	0.925 (0.451, 1.734)	73%	80%
FF/VI 100/25	BUD/FORM 400/12	0.866 (0.396, 1.664)	77%	84%
**C: change from baseline SGRQ Total score**				
**Treatment**	**Comparator**	**Mean difference, units (95% CrI)**	**Probability of non-inferiority margin (units)**	
			**2**	**3**
FF/VI 100/25	FP/SAL 500/50	−1.321 (−3.955, 1.313)	99%	>99%
FF/VI 100/25	BUD/FORM 400/12	−0.964 (−3.897, 1.970)	98%	>99%

*Note:* All stated nominal doses are mcg.

BUD = budesonide, Crl = credible interval, FORM = formoterol, FEV_1_ = forced expiratory volume in one second, FF = fluticasone furoate, FP = fluticasone propionate, S = salmeterol, SGRQ = St George’s Respiratory Questionnaire, VI = vilanterol.

*Other relevant ICS/LABA: FP/SAL 500/50 mcg and BUD/F 400/12 mcg.

†For studies in which patients were required to have an explicit exacerbation history at entry.

#### Annual moderate/severe exacerbation rates

Marked decreases relative to placebo (placebo rate normalised to 1.0) in annual moderate/severe exacerbation rates were observed for FF/VI 100/25 mcg (0.62), FP/SAL 500/50 mcg (0.66) and BUD/FORM 400/12 mcg (0.71). The CrI for FF/VI 100/25 mcg (0.285, 1.181) was very wide, resulting in its being found statistically inseparable from placebo, whereas BUD/FORM 400/12 mcg (CrI: 0.638, 0.795) and FP/SAL 500/50 mcg (CrI: 0.555, 0.790) were statistically separable from placebo (Additional file 1: e-Table S2).

Based on a non-inferiority margin representing an annual event rate ratio reduction of 10%, FF/VI 100/25 mcg demonstrated 73% probability of non-inferiority to FP/SAL 500/50 mcg and 77% probability of non-inferiority to BUD/FORM 400/12 mcg. On a less stringent 20% rate ratio margin, FF/VI 100/25 mcg has 80% probability of non-inferiority to FP/SAL 500/50 mcg and 84% probability of non-inferiority to BUD/FORM 400/12 mcg (Table 2). Significant covariate effects of age and study duration were observed: studies that enrolled older patients showed slightly lower average exacerbation rates, and reduced exacerbation rates were seen in shorter duration studies (Additional file 1: e-Table S3).

#### Change from baseline SGRQ total score

FF/VI 100/25 mcg, FP/SAL 500/50 mcg and BUD/FORM 400/12 mcg were all associated with significant improvement in estimated mean SGRQ score relative to placebo. The mean improvement observed with FF/VI 100/25 mcg (−4.599 units) exceeded the MCID of 4 units and was numerically greater than that seen with FP/SAL 500/50 mcg (−3.278) or BUD/FORM 400/12 mcg (−3.635) (Additional file 1: e-Table S2).

Based on a non-inferiority margin of 2 units (half the MCID), FF/VI 100/25 mcg demonstrated 99% probability of non-inferiority to FP/SAL 500/50 mcg and 98% probability of non-inferiority to BUD/FORM 400/12 mcg for change from baseline SGRQ score. Using a 3-unit margin, FF/VI had >99% probability of non-inferiority to both FP/SAL and BUD/FORM. No significant covariate effects on the change from baseline SGRQ Total score outcome were identified in the main analysis.

### Sensitivity analysis

A sensitivity analysis of an enlarged network (Additional file 1: e-Figure S1) was conducted for the exacerbations outcome. Six additional studies which did not require patients to have an explicit history of exacerbations were added to the primary analysis network of 15 studies. The findings of this sensitivity analysis (Additional file 1: e-Table S5) were similar to those of the primary analysis, and the CrIs for comparisons of FF/VI 100/25 mcg remained wide; thus, it was not possible to draw conclusions on non-inferiority of FF/VI 100/25 mcg on exacerbation rate from the sensitivity analysis. Separate sensitivity analyses of the exacerbation rate calculations using alternative assumptions for patients lost to follow-up reached similar conclusions to the primary analysis (Additional file 1: e-Table S6). Findings of a sensitivity analysis of the SGRQ outcome in which two studies were excluded from the network were similar to those of the primary analysis.

### Assessment of alternative modelling approaches

The findings and details of the post-hoc analysis of alternative modelling approaches – specifically, a frequentist analysis using a random effects model with fixed study and treatment effects, and a pairwise contrast analysis – are reported in Addional file 1: e-Table S7. The results of these analyses showed that, where the application of varied methodologies to the dataset was feasible, the results of analyses using these methodologies were consistent with those of the primary MTC analysis.

## Discussion

FF/VI 100/25 mcg represents the first once-daily ICS/LABA combination approved in the United States, Canada and Europe for the long-term maintenance treatment of patients with COPD. We sought to compare the clinical efficacy of FF/VI 100/25 mcg with that of the twice-daily ICS/LABA therapies FP/SAL 500/50 mcg and BUD/FORM 400/12 mcg. The comparative efficacy of FF/VI 100/25 mcg and FP/SAL 500/50 mcg has previously been investigated in head-to-head RCTs, and no significant treatment difference in terms of lung function was observed [3,4].

Using an MTC approach, we examined the probability of non-inferiority of once-daily FF/VI 100/25 mcg to corresponding strengths of twice-daily ICS/LABA combination therapies – FP/SAL 500/50 mcg and BUD/FORM 400/12 mcg – by combining data on clinical efficacy outcomes from several RCTs. The selected comparisons are presented on the basis of the robustness of the networks and the current relevance of these treatments in clinical practice. All three ICS/LABA combination therapies have been shown to be associated with improvement in these outcomes vs placebo in RCTs.

We applied a Bayesian hierarchical MTC model to combine existing data from RCTs conducted in patients with COPD, that examined at least one ICS/LABA comparator. Broad-scope searches were used to identify as many studies potentially suitable for inclusion in the MTC as possible. In an effort to maximise comparability of included data, we imposed the following MTC inclusion criteria. Studies were required to be Phase III or IV parallel-group RCTs examining at least one ICS/LABA maintenance therapy, to report usable data for at least one of the three specified MTC efficacy endpoints, to have included sufficient patients (≥10 patients aged ≥12 years) and to have been of sufficient duration (>8 weeks). Subsequently, additional endpoint-specific inclusion criteria were applied as appropriate for each endpoint. Following the modelling of the observed data, the Bayesian methodology allowed us to utilise the posterior distribution to provide a probabilistic estimate of non-inferiority for FF/VI in comparison to other ICS/LABA maintenance therapies [27].

Non-inferiority margins are not well established for COPD outcomes, and were chosen prior to the commencement of any data analysis with reference to public recommendations of the US Food and Drug Administration [28] and the European Medicines Agency (CBG-MEB, 2008) and to threshold values used for assessment of drug therapy non-inferiority in previous clinical trials in COPD. For FEV_1_, the selection of a conservative 50 mL margin was informed by the well-accepted MCID of 100–140 mL [17,29] and the use of this value in previous non-inferiority studies of tiotropium [30,31]. Non-inferiority margins reflecting exacerbation event rate ratios of 10% and 20% were selected on the basis of findings from the ISOLDE trial of ICS vs placebo [32] and a Cochrane analysis of nine ICS/LABA vs ICS studies [33] in which rate reductions of 24% and 25%, respectively, were reported. For SGRQ, non-inferiority limits of 2 and 3 scoring units were used; these margins represent half and three quarters of the accepted MCID of 4 units [18] and of the observed overall difference between ICS/LABA and placebo in the TORCH trial [34]. The selection of margins that are narrow relative to the MCID increases the difficulty of demonstrating non-inferiority of compared treatments; they are therefore regarded as conservative. It is, however, important to note that a finding of a low probability of non-inferiority does not imply lack of comparability or inferiority of the intervention.

Based on these conservative margins, the findings from our MTC analysis support previously-reported RCT findings and indicate that there is a high probability that FF/VI 100/25 mcg is non-inferior to FP/SAL 250/50 mcg and BUD/FORM 400/12 mcg on lung function (FEV_1_) and health status (SGRQ) outcomes of interest. The analysis of exacerbation rate data was inconclusive owing to insufficient data and the consequent weakness of the network for this outcome, resulting in wide CrI.

The data limitations were primarily a consequence of the limited number of RCTs evaluating the ICS/LABA treatments of interest that met the inclusion criteria and were therefore available for inclusion in the MTC. In particular, the exacerbations network was weak with respect to FF/VI, as only one 12-week study of FF/VI 100/25 mcg connected the FF/VI studies with the rest of the treatment network and this particular study reported a substantially lower annual moderate/severe exacerbation rate compared with other studies in the network. In addition, this single study link was the only study of less than 20 weeks’ duration. The model accounts for the potential effect of the study’s short length on the reported exacerbation rate; however, this inevitably impacts the informative value of this study, contributing further to the weakness of the exacerbation rates network. As a consequence, the evidence available does not allow for definitive statements regarding these comparisons on this outcome of interest.

In a sensitivity analysis of the exacerbation event rate ratio MTC, the criteria for inclusion of studies in the network were relaxed and studies which did not have an inclusion criterion of requiring an explicit history of exacerbations were included. The sensitivity analysis network included additional treatments, but the strength of the comparisons of FF/VI 100/25 mcg with corresponding doses of FP/SAL and BUD/FORM was not substantially improved and the CrIs for the non-inferiority analysis remained wide. The *post-hoc* assessment of alternative modelling approaches showed that the findings of the lung function and health status MTCs were consistent upon the application to the data of other methodologies, including a frequentist approach.

Variability in study design also impacted the precision of model estimates. The studies included in our MTC were heterogeneous in population and region. Furthermore, there is a substantial degree of inconsistency in the definitions of and measurement methodologies used to assess respiratory clinical outcomes such as FEV_1_ [35]. Some RCTs report exacerbation rates adjusted for patients lost to follow-up while other studies lack information on follow-up and report only the number of exacerbations. We attempted to address these issues in our inclusion criteria and sought to include all comparable patient populations.

An in-built assumption of the model – that could potentially be confounded by study heterogeneity with regard to aspects of the patient population and the region(s) in which the study was conducted – is that treatment effects are consistent across studies. As far as possible, over-dispersion arising as a consequence of this large study-to-study variability was observed in the distribution and accounted for through the incorporation of heterogeneity factors at the study level of the hierarchy. However, the degree to which heterogeneity could be accounted for was limited by the amount of data available and unavailability of patient-level information about covariates and outcomes.

By incorporating covariates into the model at the study level, we assessed whether study and population variability in covariates such as duration, average age and exacerbation history could have impacted the suitability for comparison of the studies included in the MTC. Age had a significant covariate effect on FEV_1_, which is to be expected since COPD is a disease characterised by progressive deterioration in lung function. Since older patients would be expected to have poorer baseline lung function than younger patients, we would expect to see smaller changes in FEV_1_ in studies recruiting a patient population with a higher average age. The observation that exacerbation rates were typically lower in shorter studies could also be due to the progressive deterioration in lung function with COPD, which is associated with increased exacerbation rates [15]. However, the finding of a significant covariate relationship of older average age with lower exacerbation rates is perhaps counterintuitive. One possible explanation is that older patients may have been selected from generally better-controlled patient populations.

MTC is an established approach to the synthesis of indirect and direct evidence to make comparisons of and draw conclusions around the relative efficacy of multiple treatments in a single model. In a previous MTC, published in 2009, data from 43 RCTs were analysed with respect to exacerbation, mortality and study withdrawal rates [36] in COPD. Relative to alternative treatment modalities, ICS/LABA combination therapy was found to have the greatest positive effect on outcomes. The results of another MTC, examining exacerbation rates using data from 26 trials, also indicated that combination therapies may offer a therapeutic advantage over monotherapies [37]. To our knowledge, our data are the first MTC findings to be reported on treatment effects on lung function and health-related quality of life in COPD.

## Conclusions

The findings of the MTC suggest that the efficacy of once-daily FF/VI 100/25 mcg in COPD is broadly comparable to that of twice-daily FP/SAL 500/50 mcg and BUD/FORM 400/12 mcg for lung function and health-related quality of life outcomes in a clinical trial setting. It should be borne in mind that the MTC findings are obtained through the analysis of outcomes from RCTs and any potential efficacy benefits that may be derived from once- vs twice-daily dosing in real-world clinical practice may not be reflected in these data.

## Additional file

Additional file 1:
**e-Tables and e-Figures.**

## Abbreviations

BUD: Budesonide
COPD: Chronic obstructive pulmonary disease
CrI: Credible interval
FORM: Formoterol
FEV_1_: Forced expiratory volume in one second
FF: Fluticasone furoate
FP: Fluticasone propionate
ICS: Inhaled corticosteroid
LABA: Long-acting beta_2_ agonist
MCID: Minimum clinically important difference
MTC: Mixed treatment comparison
RCT: Randomised controlled trial
SAL: Salmeterol
SGRQ: St George’s Respiratory Questionnaire
VI: Vilanterol
