# Supplementary material for: Once-daily fluticasone furoate/vilanterol 100/25 mcg versus twice daily combination therapies in COPD – mixed treatment comparisons of clinical efficacy
Source: Respir Res. 2015 Feb 15;16(1):25. doi: 10.1186/s12931-015-0184-8 (PMC4339422; doi:10.1186/s12931-015-0184-8)
Supplement: Additional file 1: — e-Tables and e-Figures. [file 12931_2015_184_MOESM1_ESM.pdf]

e-Table 1 Characteristics of the studies and treatment arms included in the MTC

Study	Regimen	Timepoints			Mean age	% male	% current smokers	Exac. history in past 12 mth	% pred. FEV_1
		FEV_1	Exac	SGRQ					
Anzueto, 2009	FP/SAL 250/50 BID	52	52	52	65.4	51	42	At least 1	< 50%
	SAL 50 BID	52	52	52	65.3	57	43	At least 1	< 50%
AstraZeneca, 2012	BUD/FORM 400/12 BID			12	64.5	88			50–70%
	FORM 12 BID			12	65.6	90			50–70%
Barnes, 2006	Placebo	13			63.9	74	59		50–70%
	FP/SAL 500/50 BID	13			64.9	82	63		50–70%
Calverley, 2003	Placebo		52		65.0	75	30	At least 1	<50%
	BUD/FORM 400/12 BID		52		64.0	78	33	At least 1	<50%
	BUD 400 BID		52		64.0	74	39	At least 1	<50%
	FORM 12 BID		52		63.0	75	36	At least 1	<50%
Calverley, 2003	Placebo	52	52	52	63.4	75	47	At least 1	50–70%
	SAL 50 BID	52	52	52	63.2	70	51	At least 1	50–70%
	FP 500 BID	52	52	52	63.5	70	53	At least 1	50–70%
	FP/SAL 500/50 BID	52	52	52	62.7	75	52	At least 1	50–70%
Calverley, 2007	Placebo	156		156	65.0	76	43		50–70%
	SAL 50 BID	156		156	65.1	76	43		50–70%

Study	Regimen	Timepoints			Mean age	% male	% current smokers	Exac. history in past 12 mth	% pred. FEV_1
		FEV_1	Exac	SGRQ					
	FP 500 BID	156		156	65.0	75	43		50–70%
	FP/SAL 500/50 BID	156		156	65.0	75	43		50–70%
Calverley, 2010	BDP (extra fine)/FORM 200/12 BID	48	48	48	63.0	79	39	At least 1	<50%
	BUD/FORM 400/12 BID	48	48	48	64.1	82	36	At least 1	<50%
	FORM 12 BID	48	48	48	63.7	81	37	At least 1	<50%
Cazzola, 2000	SAL 50 BID	12			64.6	90			>70%
	FP/SAL 250/50 BID	12			63.7	100			>70%
	FP/SAL 500/50 BID	12			64.2	85			>70%
	SAL 50 BID + theophylline	12			64.7	90			>70%
Cazzola, 2007	FP/SAL 500/50 BID	12			64.4	87	93		<50%
	TIO 18 QD	12			66.1	93	83		<50%
	FP/SAL 500/50 BID + TIO18 QD	12			66.9	87	80		<50%
Ferguson, 2008 (SCO40043)	FP/SAL 250/50 BID	52	52	52	64.9	58	40	At least 1	<50%
	SAL 50 BID	52	52	52	65.0	52	38	At least 1	<50%
GlaxoSmithKline, 2005 (SCO30002)*	Placebo		54		65.7	0.80	0.36		50–70%
	FP/SAL 500/50 BID		54		63.9	0.84	0.42		50–70%
	FP 500 BID		54		64.6	0.83	0.42		50–70%

Study	Regimen	Timepoints			Mean age	% male	% current smokers	Exac. history in past 12 mth	% pred. FEV_1
		FEV_1	Exac	SGRQ					
GlaxoSmithKline, 2006 (SCO100470)	SAL 50 BID	24		24	63.7	77	44		>70%
	FP/SAL 250/50 BID	24		24	63.5	78	42		>70%
GlaxoSmithKline, 2010 (Hagedorn 2013)	FP/SAL 500/50 BID	52	52	52	65.5	69	31	At least 2	<50%
	FP/SAL 500/50 BID	52	52	52	64.2	72	26	At least 2	<50%
GlaxoSmithKline, 2011 (ADC112355)	Placebo	16			63.5	74	59		>70%
	FP/SAL 250/50 BID	16			63.6	68	63		>70%
Dransfield, 2013 (HZC102970)	VI 25 QD	52	52		63.6	57	46	At least 1	50–70%
	FF/VI 50/25 QD	52	52		63.7	56	47	At least 1	50–70%
	FF/VI 100/25 QD	52	52		64.0	55	46	At least 1	50–70%
	FF/VI 200/25 QD	52	52		63.5	53	45	At least 1	50–70%
Agusti, 2013 (HZC113107)	FF/VI 100/25 QD	12	13	12	63.0	80	47	At least 1	50–70%
	FP/SAL 500/50 BID	12	13	12	62.9	84	37	At least 1	50–70%
Hanania, 2003	Placebo	24			65.0	68	47		50–70%
	SAL 50 BID	24			64.0	58	51		50–70%
	FP 250 BID	24			63.0	66	48		50–70%
	FP/SAL 250/50 BID	24			63.0	61	43		50–70%
Dransfield, 2013 (HZC102871)	VI 25 QD	52	52		63.6	58	43	At least 1	50–70%
	FF/VI 50/25 QD	52	52		63.6	60	42	At least 1	50–70%

Study	Regimen	Timepoints			Mean age	% male	% current smokers	Exac. history in past 12 mth	% pred. FEV_1
		FEV_1	Exac	SGRQ					
	FF/VI 100/25 QD	52	52		63.6	57	43	At least 1	50–70%
	FF/VI 200/25 QD	52	52		63.8	62	41	At least 1	50–70%
HZC112352	FF/VI 100/25 QD	12			61.6	70	47		50–70%
	FP/SAL 250/50 BID	12			61.7	66	52		50–70%
HZC113109	FF/VI 100/25 QD	12			61.1	63	54		50–70%
	FP/SAL 250/50 BID	12			61.2	65	55		50–70%
Kardos, 2007	FP/SAL 500/50 BID	44	44	44	63.8	74	41	At least 2	<50%
	SAL 50 BID	44	44	44	64.0	78	44	At least 2	<50%
Kerwin/HZC112206, 2013	Placebo	24			62.1	68	54		50–70%
	FF 100 QD	24			62.7	64	54		50–70%
	VI 25 QD	24			63.4	68	54		50–70%
	FF/VI 50/25 QD	24			62.8	66	54		50–70%
	FF/VI 100/25 QD	24			62.3	67	54		50–70%
Mahler, 2002	Placebo	24			64.0	75	54		50–70%
	SAL 50 BID	24			63.5	64	46		50–70%
	FP 500 BID	24			64.4	61	46		50–70%
	FP/SAL 500/50 BID	24			61.9	62	46		50–70%

Study	Regimen	Timepoints			Mean age	% male	% current smokers	Exac. history in past 12 mth	% pred. FEV_1
		FEV_1	Exac	SGRQ					
Martinez / HZC112207, 2013	Placebo	24			61.9	74	53		50–70%
	FF 100 QD	24			61.8	74	56		50–70%
	VI 25 QD	24			61.2	74	55		50–70%
	FF/VI 100/25 QD	24			61.9	71	53		50–70%
	FF/VI 200/25 QD	24			61.1	67	55		50–70%
	FF 200 QD	24			61.8	74	55		50–70%
Perng, 2009	FP/SAL 500/50 BID	12		12	72.0	94	57		>70%
	FP 500 BID + TIO 18 QD	12		12	74.1	100	60		>70%
	TIO 18 QD	12		12	74.3	94	65		>70%
Rennard, 2009	Placebo	52		52	62.9	65	44	At least 1	<50%
	BUD/FORM 400/12 BID	52		52	63.2	62	39	At least 1	<50%
	BUD/FORM 200/12 BID	52		52	63.6	63	42	At least 1	<50%
	FORM 12 BID	52		52	62.9	65	45	At least 1	<50%
Sharafkhaneh, 2012	BUD/FORM 400/12 BID	52	52	52	63.8	64	32	At least 1	<50%
	BUD/FORM 200/12 BID	52	52	52	62.8	65	33	At least 1	<50%
	FORM 12 BID	52	52	52	62.5	57	34	At least 1	<50%

Study	Regimen	Timepoints			Mean age	% male	% current smokers	Exac. history in past 12 mth	% pred. FEV_1
		FEV_1	Exac	SGRQ					
Szafranski, 2003	Placebo		52	52	65.0	83	34	At least 1	<50%
	BUD/FORM 400/12 BID		52	52	64.0	76	30	At least 1	<50%
	BUD 200 BID		52	52	64.0	80	36	At least 1	<50%
	FORM 6 BID		52	52	63.0	76	38	At least 1	<50%
Tashkin, 2008	Placebo	26		26	63.2	69	40	At least 1	<50%
	BUD/FORM 400/12 BID	26		26	63.1	68	44	At least 1	<50%
	BUD/FORM 200/12 BID	26		26	63.6	64	45	At least 1	<50%
	BUD/FORM 400/12 BID	26		26	63.7	74	42	At least 1	<50%
	BUD 400 BID	26		26	63.4	68	43	At least 1	<50%
	FORM 12 BID	26		26	63.5	66	42	At least 1	<50%
Tashkin, 2012	Placebo	26		26	58.8	78	48		50–70%
	MMF/FORM 200/10 BID	26		26	60.4	75	47		50–70%
	MMF/FORM 400/10 BID	26		26	59.4	77	51		50–70%
	MMF 400 BID	26		26	60.2	78	49		50–70%
	FORM 12 BID	26		26	59.7	74	48		50–70%
Wedzicha, 2008	FP/SAL 500/50 BID	104	104	104	64.0	81	38	At least 1	<50%
	TIO 18 QD	104	104	104	65.0	84	38	At least 1	<50%
Wouters, 2005	SAL 50 BID	52	52	52	64.0	75	35	At least 2	50–70%
	FP/SAL 500/50 BID	52	52	52	63.0	73	39	At least 2	50–70%

Study	Regimen	Timepoints			Mean age	% male	% current smokers	Exac. history in past 12 mth	% pred. FEV_1
		FEV_1	Exac	SGRQ					
Zheng, 2007	Placebo			24	66.6	86	23		50–70%
	FP/SAL 500/50 BID			24	66.0	91	21		50–70%
Zhong, 2012	BUD/FORM 400/12 BID		24	24	65.7	98		At least 1	<50%
	BUD 400 BID		24	24	64.7	92		At least 1	<50%

* Study not included in primary analysis

Note: All stated doses are μg. Delivered dose is given for FF/VI at the strength licenced in Europe and the United States for the treatment of COPD and for BUD/FORM; for all other treatments, nominal doses are given.

BDP = beclomethasone, BID = twice daily, BUD = budesonide, FEV_1 = forced expiratory volume in one second, FF = fluticasone furoate, FORM = formoterol, FP = fluticasone propionate, MMF = mometasone furoate, QD = once daily, SAL = salmeterol, SGRQ = St George's Respiratory Questionnaire, TIO = tiotropium; VI = vilanterol

e-Table 2 Results of mixed treatment comparisons by ICS/LABA treatment (primary analysis population; full covariate model, if available)

A: change from baseline FEV_1, L; B: annual rate of exacerbations; C: change from baseline SGRQ Total score

A

	Change from baseline FEV_1, L	
**Treatment (mcg)**	**Mean change from baseline (SD)**	**Mean difference from placebo (95% CrI)**
Placebo	–0.123 (0.039)	-
FF/VI 100/25	0.028 (0.038)	0.151 (0.126, 0.175)
FF/VI 200/25	0.024 (0.039)	0.147 (0.117, 0.177)
FF/VI 50/25	0.022 (0.038)	0.145 (0.115, 0.175)
FP/SAL 500/50	0.005 (0.040)	0.128 (0.110, 0.146)
FP/SAL 250/50	0.004 (0.040)	0.127 (0.104, 0.149)
BUD/FORM 400/12	0.001 (0.042)	0.125 (0.101, 0.147)
MMF/FORM 400/10	–0.001 (0.041)	0.122 (0.087, 0.156)
BDP/FORM 200/12	–0.004 (0.047)	0.119 (0.070, 0.167)
BUD/FORM 200/12	–0.010 (0.042)	0.113 (0.088, 0.138)
MMF/FORM 200/10	–0.026 (0.044)	0.097 (0.062, 0.130)

B

Treatment (mcg)	Annual exacerbation rate<br><br>Mean event rate ratio to placebo (95% CrI)
Placebo	1
FF/VI 100/25	0.617 (0.285, 1.181)
FF/VI 200/25	0.644 (0.301, 1.225)
FF/VI 50/25	0.700 (0.324, 1.336)
FP/SAL 500/50	0.664 (0.555, 0.790)
FP/SAL 250/50	0.601 (0.487, 0.731)
BUD/FORM 400/12	0.714 (0.638, 0.795)
MMF/FORM 400/10	–
BDP/FORM 200/12	0.745 (0.540, 1.004)
BUD/FORM 200/12	0.711 (0.585, 0.850)
MMF/FORM 200/10	–

C

Treatment (mcg)	Change from baseline SGRQ Total score, units	
	**Mean change from baseline (SD)**	**Mean difference from placebo (95% CrI)**
Placebo	0.874 (2.311)	–
FF/VI 100/25	–3.725 (2.640)	–4.599 (–7.362, –1.800)
FF/VI 200/25	–	–
FF/VI 50/25	–	–
FP/SAL 500/50	–2.404 (2.302)	–3.278 (–4.127, –2.435)
FP/SAL 250/50	–1.349 (2.348)	–2.222 (–3.562, –0.880)
BUD/FORM 400/12	–2.761 (2.404)	–3.635 (–4.647, –2.614)
MMF/FORM 400/10	–2.526 (2.462)	–3.399 (–5.029, –1.748)
BDP/FORM 200/12	–2.356 (2.540)	–3.229 (–5.505, –0.938)
BUD/FORM 200/12	–2.513 (2.440)	–3.386 (–4.586, –2.140)
MMF/FORM 200/10	–2.729 (2.574)	–3.603 (–5.322, –1.888)

Note: All stated doses are µg

BDP = beclomethasone dipropionate, BID = twice daily, BUD = budesonide, CrI = credible interval, FEV_1 = forced expiratory volume in one second, FORM = formoterol, FF = fluticasone furoate, FP = fluticasone propionate, MMF = mometasone furoate, QD = once daily, SAL = salmeterol, SD = standard deviation, SGRQ = St George's Respiratory Questionnaire, VI = vilanterol

e-Table 3 Summary of findings of covariate analysis by outcome of interest (primary analysis population; full covariate model, if available)

A: change from baseline FEV_1, L; B: annual rate of exacerbations; C: change from baseline SGRQ Total score

A

**Covariate**	**Mean (SD)**	**95% CrI**
Age (age – 60)	–0.003* (0.001)	–0.005, –0.001
Male (proportion males – 0.75)	–0.010 (0.072)	–0.151, 0.132
Smoking status (proportion smokers – 0.5)	–0.082 (0.089)	–0.256, 0.092
FEV_1 % predicted at baseline		
>70%	0.036 (0.039)	–0.040, 0.112
50% ≤ x ≤ 70%	Reference	Reference
<50%	0.047 (0.038)	–0.027, 0.121
Exacerbation history		
≥1 prev. year	Reference	Reference
≥2 prev. year	0.017 (0.033)	–0.048, 0.082
No data	0.020 (0.013)	–0.006, 0.045
Study length		
≤20 weeks	0.097 (0.050)	0.000, 0.194
>0–≤40 weeks	0.098 (0.032)	–0.036, 0.161
>40–≤60 weeks	Reference	Reference
>60 weeks	–0.009 (0.020)	–0.048, 0.030

*Significant

B

Covariate	Mean (SD)	Rate Ratio (SD)	Rate Ratio 95% CrI
Age (age – 60)	–0.047 (0.019)	0.954* (0.018)	0.919, 0.990
Male (proportion males – 0.75)	0.452 (0.587)	1.868 (1.165)	0.497, 4.969
Smoking status (proportion smokers – 0.5)	0.336 (0.941)	2.133 (2.173)	0.221, 8.858
FEV_1 % predicted at baseline			
>70%	–0.239 (0.238)	0.812 (0.183)	0.495, 1.260
50% ≤ x ≤ 70%	Reference	Reference	Reference
<50%	–	–	–
Exacerbation history			
≥1 prev. year	Reference	Reference	Reference
≥2 prev. year	0.052 (0.326)	1.108 (0.349)	0.556, 1.995
Study length			
≤20 weeks	–2.453 (0.621)	0.104* (0.070)	0.026, 0.290
>20–≤40 weeks	–0.013 (0.576)	1.215 (0.922)	0.328, 3.131
>40–≤60 weeks	Reference	Reference	Reference
>60 weeks	0.651 (0.349)	2.027 (0.623)	0.968, 3.799

*Significant

C

**Covariate**	**Mean (SD)**	**95% CrI**
Age (age – 60)	–0.178 (0.270)	–0.707, 0.352
Male (proportion males – 0.75)	–2.706 (4.534)	–11.592, 6.180
Smoking status (proportion smokers – 0.5)	–1.775 (5.856)	–13.252, 9.703
FEV_1 % predicted at baseline		
>70%	–3.742 (3.676)	–10.947, 3.463
50% ≤ x ≤ 70%	Reference	Reference
<50%	–0.265 (2.066)	–4.314, 3.785
Exacerbation history		
≥1 prev. year	Reference	Reference
≥2 prev. year	1.891 (2.217)	–2.455, 6.237
No data	–2.549 (3.418)	–9.248, 4.149
Study length		
≤20 weeks	0.665 (2.811)	–4.845, 6.175
>20–≤40 weeks	–1.881 (2.407)	–6.599, 2.838
>40–≤60 weeks	Reference	Reference
>60 weeks	2.142 (2.964)	–3.668, 7.952

CrI = credible interval, FEV_1 = forced expiratory volume in one second, SD = standard deviation

eTable 4 Posterior probability of non-inferiority for FF/VI 100/25 mcg versus other relevant ICS/LABA* (model with study duration covariate only)

A: change from baseline FEV_1, L; B: annual rate of exacerbations; C: change from baseline SGRQ Total score

*Other relevant ICS/LABA: FP/SAL 500/50 mcg and BUD/F 400/12 mcg

A

Treatment	Comparator	Mean difference, L (95% CrI)	Probability of non-inferiority Threshold (change from baseline) 50 ml
FF/VI 100/25	FP/SAL 500/50	0.016 (–0.007, 0.039)	>99%
FF/VI 100/25	BUD/FORM 400/12	0.020 (–0.011, 0.051)	>99%

B

Treatment	Comparator	Hazard Ratio (95% CrI)	Probability of non-inferiority Threshold (event rate ratio)	
			0.10	0.20
FF/VI 100/25	FP/SAL 500/50	0.871 (0.499, 1.536)	86%	91%
FF/VI 100/25	BUD/FORM 400/12	0.823 (0.460, 1.472)	90%	93%

* Studies in which patients were required to have an explicit exacerbation history at entry

C

			Probability of non-inferiority	
			Threshold (units)	
**Treatment**	**Comparator**	**Mean difference (95% CrI)**	**2**	**3**
FF/VI 100/25	FP/SAL 500/50	–1.120 (–3.398, 1.158)	>99%	>99%
FF/VI 100/25	BUD/FORM 400/12	–0.782 (–3.342, 1.779)	98%	>99%

Note: All stated doses are μg.

BUD = budesonide, CrI = credible interval, FORM = formoterol, FF = fluticasone furoate, FP = fluticasone propionate, SAL = salmeterol, VI = vilanterol

e-Table 5 Posterior probability of non-inferiority for FF/VI 100/25 mcg versus other relevant ICS/LABA combination therapies, sensitivity analysis (full covariate model)

A: annual rate of exacerbations; B: change from baseline SGRQ Total score

*Other relevant ICS/LABA: FP/SAL 500/50 mcg and BUD/F 400/12 mcg.

A

Treatment	Comparator	Rate Ratio (95% CrI)	Probability of non-inferiority margin (event rate ratio)	
			**0.10**	**0.20**
FF/VI 100/25	FP/SAL 500/50	0.889 (0.494, 1.508)	82%	89%
FF/VI 100/25	BUD/FORM 400/12	0.913 (0.508, 1.551)	79%	87%

B

Treatment	Comparator	Mean difference, units (95% CrI)	Probability of non-inferiority margin (units)	
			**2**	**3**
FF/VI 100/25	FP/SAL 500/50	–1.891 (–4.691, 0.910)	>99%	>99%
FF/VI 100/25	BUD/FORM 400/12	–1.564 (–4.624, 1.496)	99%	>99%

Note: All stated doses are μg.

BUD = budesonide, CrI = credible interval, FORM = formoterol, FF = fluticasone furoate, FP = fluticasone propionate, SAL = salmeterol, VI = vilanterol

e-Table 6 Posterior probability of non-inferiority for FF/VI 100/25 mcg versus other relevant ICS/LABA combination therapies on annual rate of moderate/severe exacerbations, sensitivity analysis (full covariate model)

Sensitivity analysis in which the assumptions made in estimating person-years of follow-up for patients lost to follow-up in studies for which these data were unavailable were varied from the base case assumption of 50% of potential follow-up time to A: 25% and B: 75%.

*Other relevant ICS/LABA: FP/SAL 500/50 mcg and BUD/F 400/12 mcg.

A

Treatment	Comparator	Rate Ratio (95% CrI)	Probability of non-inferiority margin (event rate ratio) 0.10	0.20
FF/VI 100/25	FP/SAL 500/50	0.838 (0.393, 1.932)	83%	87%
FF/VI 100/25	BUD/FORM 400/12	0.778 (0.348, 1.799)	86%	89%

B

Treatment	Comparator	Rate Ratio (95% CrI)	Probability of non-inferiority margin (event rate ratio) 0.10	0.20
FF/VI 100/25	FP/SAL 500/50	0.784 (0.407, 1.653)	87%	91%
FF/VI 100/25	BUD/FORM 400/12	0.708 (0.351, 1.507)	91%	93%

e-Table 7 Outcomes of assessment of alternative modelling approaches ([1] full covariate random study effects model, [2] no covariates random effects contrast based model, [3] full covariate fixed study effects model)

A: change from baseline FEV_1, L; B: exacerbations; C: change from baseline SGRQ Total score

A

Treatment	Comparator	Mean difference [1] (95% CrI)	Probability of non-inferiority Pr (A~B|50 ml)	geMTC Mean difference (95% CrI) [2]	Fixed study effects Mean difference (95% CrI) [3]	p-value [3]
FF/VI 100/25	FP/SAL 500/50	0.023 (-0.002, 0.048)	>99%	0.01 (-0.02, 0.05)	0.023 (-0.009, 0.063)	0.140
FF/VI 100/25	BUD/FORM 400/12	0.027 (-0.007, 0.061)	>99%	0.04 (0.00, 0.09)	0.020 (-0.027, 0.067	0.397

B

Treatment	Comparator	HR (95% CrI) [1]	Probability of non-inferiority Pr (A~B |0.10)	Pr (A~B |0.20)	geMTC Mean difference (95% CrI) (no covariates) [2]	Fixed study effects Rate ratio (95% CrI) [3]	p-value [3]
FF/VI 100/25	FP/SAL 500/50	0.925 (0.451, 1.734)	73%	80%	-0.01 (-0.38, 0.35)	0.901 (0.296, 2.745)	0.854
FF/VI 100/25	BUD/FORM 400/12	0.866 (0.396, 1.664)	77%	84%	0.00 (-0.50, 0.48)	0.851 (0.269, 2.689)	0.784

C

Treatment	Comparator	Mean difference (95% CrI) [1]	Probability of non-inferiority Pr (A~B|2)	Pr (A~B|3)	geMTC Mean difference (95% CrI) (no covariates) [2]	Fixed study effects Mean difference (95% CrI) [3]	p-value [3]
FF/VI 100/25	FP/SAL 500/50	-1.339 (-4.182, 1.505)	99%	>99%	-1.34 (-5.03, 2.25)	-1.723 (-5.041, 1.595)	0.309
FF/VI 100/25	BUD/FORM 400/12	-1.085 (-4.213, 2.043)	97%	99%	-1.33 (-5.68, 2.83)	0.507 (-2.417, 3.431)	0.734

Note: All stated doses are µg.

BUD = budesonide, CrI = credible interval, FORM = formoterol, FF = fluticasone furoate, FP = fluticasone propionate, ; HR: = hazard ratio, SAL = salmeterol; VI = vilanterol

e-Figure 1 Networks of study treatments, by outcome of interest (annual rate of exacerbations, sensitivity analysis population)

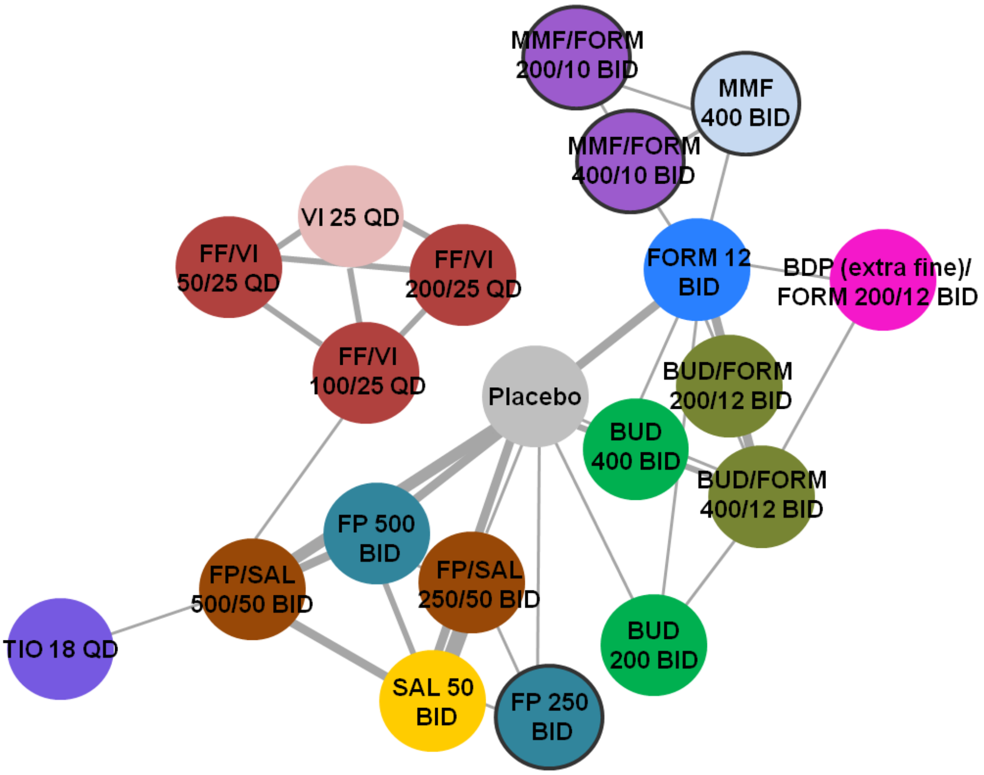

Note: All stated nominal doses are µg. Connecting lines represent studies included in the model that directly compare the two treatments. The thickness of the line is proportional to the number of studies comparing the two treatments. Treatments outlined in dark grey are included in the sensitivity analysis only (i.e. not in the primary analysis).

BDP = beclomethasone dipropionate, BID = twice daily, BUD = budesonide, FORM = formoterol, FF = fluticasone furoate, FP = fluticasone propionate, MMF = mometasone furoate, QD = once daily, SAL = salmeterol, THEO = theophylline, TIO = tiotropium, VI = vilanterol

Data sources used in systematic literature search to identify shortlisted studies

Data sources	
**Databases**	Clinical Publication Databases<br>• Medline (OvidSP)<br>• Medline In-Process Citations & Daily Update (OvidSP)<br>• Embase (OvidSP)<br>• Cochrane Database Of Systematic Reviews (CDSR) (Wiley)<br>• Cochrane Central Register of Controlled Trials (CENTRAL) (Wiley)<br>• Database of Abstracts of Reviews of Effects (DARE) (Wiley)<br>• Health Technology Assessment Database (HTA) (Wiley)<br>• NIHR Health Technology Assessment Programme (Internet)<br>• PROSPERO (International Prospective Register of Systematic Reviews) (Internet) http://www.crd.york.ac.uk/prospero/<br><br>Clinical trials registers<br>• NIH Clinicaltrials.gov (Internet) http://www.clinicaltrials.gov/<br>• Current Controlled Trials (Internet) http://www.controlled-trials.com/<br>• WHO International Clinical Trials Registry Platform (ICTRP) (Internet) http://www.who.int/ictrp/en/<br>• European Medicines Agency European Public Assessment Reports (EMA EPARs) (http://www.ema.europa.eu/htms/human/epar/a.htm)<br>• FDA website<br>• Conference abstracts from COPD conferences (American Thoracic Society (ATS), European Respiratory Society (ERS), American College of Chest Physicians (ACCP), from 2010).

Study selection for primary analyses of outcomes of interest

Of the 59 studies identified by the systematic literature search, 22 were excluded as follows:

- 11 did not report results for any of the efficacy outcomes of interest
- 7 were of insufficient duration, i.e. ≤8 weeks
- 2 were excluded because of small study populations and lack of usable data
- 1 was a small-scale (N<10) exploratory study with undefined primary efficacy endpoint
- 1 was excluded as it was a Phase II study

Further to the above, two replicate studies whose data was also reported in a pooled analysis were identified by the literature search. For simplicity and consistency, the pooled data were included in the analysis rather than the individual studies. Thirty six studies (including the pooled analysis) were therefore considered for inclusion in the three outcome analyses. Of these, 33 were included in at least one of the three main MTC analyses.

Change from baseline in FEV_1

Of the aforementioned 36 studies, 8 were excluded from the FEV_1 analysis because change from baseline in FEV_1 was not reported. Twenty eight studies were therefore included in the FEV_1 network.

Annual rate of moderate/severe exacerbations

Ten of the 36 studies were excluded from the exacerbations analysis because usable exacerbation data could not be extracted or calculated. Eleven studies were excluded from the main analysis because patients did not have a documented history of exacerbations, or exacerbation history data were not collected or reported. The exacerbation analysis therefore comprised data from 15 studies. Of the 11 studies that were excluded because of lack of explicit history of exacerbations prior to study start, six were examined in a sensitivity analysis of the exacerbations model in which the exacerbation history covariate was not considered.

Change from baseline in SGRQ

Of the 36 studies under consideration, 20 were included in the SGRQ analysis. Of those that were excluded, 12 did not make any reference to SGRQ; the remaining four did not report quantitative findings, did not report change from baseline or were affected by confounding factors that limited the applicability of the findings.
